# Stimulation of Alpha7 Nicotinic Acetylcholine Receptor Attenuates Nicotine-Induced Upregulation of MMP, MCP-1, and RANTES through Modulating ERK1/2/AP-1 Signaling Pathway in RAW264.7 and MOVAS Cells

**DOI:** 10.1155/2017/2401027

**Published:** 2017-11-16

**Authors:** Liping Liu, Hongxian Wu, Qunan Cao, Zhenzhen Guo, Anmin Ren, Qiuyan Dai

**Affiliations:** ^1^Department of Cardiology, Shanghai General Hospital of Nanjing Medical University, Shanghai Jiaotong University, Shanghai, China; ^2^Department of Cardiology, Yancheng First People's Hospital, The Fourth Affiliated Hospital of Nantong Medical University, Jiangsu, China

## Abstract

Vagus nerve stimulation through alpha7 nicotine acetylcholine receptors (*α*7-nAChR) signaling had been demonstrated attenuation of inflammation. This study aimed to determine whether PNU-282987, a selective *α*7-nAChR agonist, affected activities of matrix metalloproteinase (MMP) and inflammatory cytokines in nicotine-treatment RAW264.7 and MOVAS cells and to assess the underlying molecular mechanisms. RAW264.7 and MOVAS cells were treated with nicotine at different concentrations (0, 1, 10, and 100 ng/ml) for 0–120 min. Nicotine markedly stimulated the phosphorylation of extracellular signal-regulated kinase1/2 (ERK1/2) and c-Jun in RAW264.7 cells. Pretreatment with U0126 significantly suppressed phosphorylation of ERK1/2 and further attenuated nicotine-induced activation of c-Jun and upregulation of MMP-2, MMP-9, monocyte chemotactic protein- (MCP-) 1, and regulated upon activation normal T cell expressed and secreted (RANTES). Similarly, nicotine treatment also increased phosphorylation of c-Jun and expressions of MMP-2, MMP-9, MCP-1, and RANTES in MOVAS cells. When cells were pretreated with PNU-282987, nicotine-induced activations of ERK1/2 and c-Jun in RAW264.7 cells and c-Jun in MOVAS cells were effectively inhibited. Furthermore, nicotine-induced secretions of MMP-2, MMP-9, MCP-1, and RANTES were remarkably downregulated. Treatment with *α*7-nAChR agonist inhibits nicotine-induced upregulation of MMP and inflammatory cytokines through modulating ERK1/2/AP-1 signaling in RAW264.7 cells and AP-1 in MOVAS cells, providing a new therapeutic for abdominal aortic aneurysm.

## 1. Introduction

Abdominal aortic aneurysm (AAA) is defined as a dilatation of abdominal aorta (almost exclusively infrarenal aorta) that reaches a diameter of 30 mm or more [1]. AAA often remains asymptomatic until it ruptures with a mortality rate of 80% [2]. Rather than a consequence of advanced atherosclerosis, AAA is a local representation of systemic vascular disease, with a differential molecular and cellular profile [3, 4]. AAA is characterized by dilatation of all layers of the arterial wall as a result of inflammatory infiltration, loss of elastin, and SMC apoptosis [3, 5]. Experimental data and studies of human AAA have identified that extensive inflammatory infiltrate in the media and adventitia is composed of macrophages and lymphocytes, which secret multiple cytokines, involving in the pathological process of AAA [6–8]. Matrix metalloproteinases (MMPs), derived from vascular smooth muscle cells (VSMCs) and macrophages, are secreted into the extracellular matrix and result in the destruction of elastin and weakening of the aortic wall [5].

Smoking is a very strong modifiable risk factor for AAA [9–13]. In a recent report of enrolling 18,782 participants aged ≥ 65 years in Southern Community Cohort Study (SCCS), over a median follow-up of 4.94 years, 40% of AAA were current smokers, and another 40% were former smokers [9]. Smoking, especially current smoking, significantly increased the risk of AAA (current: HR 5.55, 95% confidence interval (CI) 3.67 to 8.40; former: HR 1.91, 95% CI 1.27 to 2.87). Increased duration of smoking and daily cigarette quantity contribute to a higher risk of incident AAA, and the effects are dose dependent [11, 12]. Moreover, rates of AAA expansion and the risk of rupture are markedly elevated in those who continued to smoke. In a meta-analysis by Sweeting et al. [13], AAA growth rates were increased by about one-sixth and rupture rates were doubled in current smokers as compared to ex- or never smokers. Although prevalence, incidence, and mortality have declined because of a reduced smoking rate [14, 15], AAA remains the 16th leading cause of death in the USA among those aged above 65 years [9], thus highlighting the need for more efforts to improve the treatment and prognosis. Our previous study showed that c-Jun N-terminal kinase (JNK) inhibitor attenuated nicotine plus AngII-induced AAA formation by suppressing MMP-9, MMP2, monocyte chemotactic protein- (MCP-) 1, and regulated upon activation normal T cell expressed and secreted (RANTES) secretion from macrophages and VSMCs, suggesting that JNK was a signaling molecule in the pathogenesis of nicotine plus AngII-induced AAA [16]. However, distal signaling molecules or nuclear transcription factors involving in the signal transduction remain elusive.

Vagus nerve stimulation through alpha7 nicotine acetylcholine receptors (*α*7-nAChR) signaling, known as the cholinergic anti-inflammatory pathway, had been demonstrated attenuation of inflammation and improvement of inflammatory diseases, such as sepsis, pancreatitis, haemorrhagic shock and ischaemia/reperfusion, and postoperative ileus in experimental models [17]. Recent studies showed that vagus nerve stimulation may also have beneficial role in cardiovascular diseases through modulation of cytokine levels, which is dependent from heart rate variability [18]. Activation of *α*7-nAChR had been found to prevent the development and progression of AAA in CaCl_2_ application mouse model in association with reduced inflammation and matrix degradation [19]. In the present study, we determined whether PNU-282987, a selective *α*7-nAChR agonist, affected activities of MMP-2 and MMP-9 and expressions of inflammatory cytokines MCP-1 and RANTES in nicotine treatment RAW264.7 and MOVAS cells.

## 2. Material and Methods

### 2.1. Reagents and Antibodies

Nicotine and PNU-282987 were obtained from Sigma-Aldrich. U0126 (a highly selective inhibitor of MEK1 and MEK2, which are kinase of ERK1/2) was from Cell Signaling Technology (CST). Monoclonal rabbit antiextracellular signal-regulated kinase1/2 (ERK1/2), antiphosphorylated ERK1/2, anti-c-Jun, antiphosphorylated c-Jun, antinuclear factor-*κ*B (NF-*κ*B) p65, and antiphosphorylated NF-*κ*B p65 were from CST. Rat polyclonal antibody to MMP2, MCP-1, RANTES, and GAPDH were from Abcam. Mouse monoclonal anti-MMP9 and MMP2 were from Santa Cruz Biotechnology.

### 2.2. Cell Culture and Treatment

A mouse macrophage cell line (RAW264.7 cells) and mouse aortic smooth muscle cell (SMC) line (MOVAS cells) were bought from the American Type Culture Collection (Manassas, VA). Cells were cultured in high-glucose Dulbecco's Modified Eagle Medium (DMEM, HyClone, Logan, UT, USA) supplemented with 10% fetal bovine serum (FBS, Gibco, Australia) at 37°C in a humidified, 5% CO_2_ atmosphere. After starved in serum-free medium overnight, cells were treated with nicotine or PNU-282987. Additional experiments were performed with RAW264.7 cells pretreated with U0126 or PNU-282987 and MOVAS cells preincubated with PNU-282987 prior to exposing to nicotine.

### 2.3. Western Blot Analysis

Cells were washed twice with ice-cold phosphate-buffered saline (PBS), lysed with RIPA Lysis Buffer (Beyotime Institute of Biotechnology, Jiangsu, China), supplemented with phosphatase inhibitor (Sangon Biotechnology, Shanghai, China) and PMSF (Roche, Molecular Biochemicals, Mannheim, Germany), centrifuged, and quantified with a BCA protein assay kit (Beyotime Institute of Biotechnology, Jiangsu, China) according to the manufacturer's instructions. Aliquots of total protein were separated by 10% sodium dodecyl sulfate polyacrylamide gel electrophoresis (SDS-PAGE) and then transferred to polyvinylidene fluoride (PVDF) membranes (Bio-Rad Laboratories, Hercules, USA). Membranes were blocked in 5% nonfat milk/TBST (25 mM Tris-HCl, 150 mM NaCl, and 0.1% Tween-20; pH 7.4) for 1 h at room temperature and subsequently incubated with primary antibodies overnight at 4°C. After three washes, membranes were incubated with horseradish peroxidase- (HRP-) conjugated goat anti-rabbit or -mouse IgG for 1 h, washed again with TBST, and subsequently visualized using West-Pico ECL kit (Pierce, Rockford, USA).

### 2.4. Real-Time Reverse Transcription Polymerase Chain Reaction (RT-PCR)

Total RNA was extracted from RAW264.7 and MOVAS cells using TRIZOL reagent (Invitrogen, Carlsbad, USA) and reversely transcribed into cDNA with PrimeScript RT Master Mix (Takara, Kusatsu, Japan) according to the manufacturer's protocol. Quantitative RT-PCR was performed using SYBR Premix Ex Taq (Takara, Kusatsu, Japan) on the Applied Biosystems ViiA™ 7 Real-Time PCR System. The specific primers used in the present study were as follows: MMP-9: 5′-GCCCTGGAACTCACACGACA-3′ (Forward) and 5′-TTGGAAACTCACACGCCAGAAG-3′ (Reverse); MMP-2: 5′-GATAACCTGGATGCCGTCGTG-3′ (Forward) and 5′-GGTGTGCAGCGATGAAGATGATA-3′ (Reverse); MCP-1: 5′-GCATCCACGTGTTGGCTCA-3′ (Forward) and 5′-CTCCAGCCTACTCATTGGGATCA-3′ (Reverse); RANTES 5′-GAAAGAACCGCCAAGTGTGT-3′ (Forward) and 5′-GCAAGCAGAAACAGGCAAAT-3′ (Reverse); and GAPDH: 5′-GTATGACTCTACCC ACGGCAAGT-3′ (Forward) and 5′-TTCCCGTTGATGACCAGCTT-3′ (Reverse). The cycle threshold (Ct) obtained for target gene expression was normalized to GAPDH, and the relative expression was calculated using the 2^−ΔΔ^Ct methods.

### 2.5. Statistical Analysis

Data were presented as mean ± standard deviations (SD). Densitometric analysis of protein bands in the Western blot was performed using ImageJ software from the National Institutes of Health. Data were analyzed by one-way ANOVA followed by the Dunnett's test. Statistical analysis was conducted using SPSS version 22. *P* < 0.05 was considered statistically significant.

## 3. Results

### 3.1. Nicotine Stimulated Phosphorylation of ERK1/2 and c-Jun in RAW264.7 Cells

**Figure 1:**
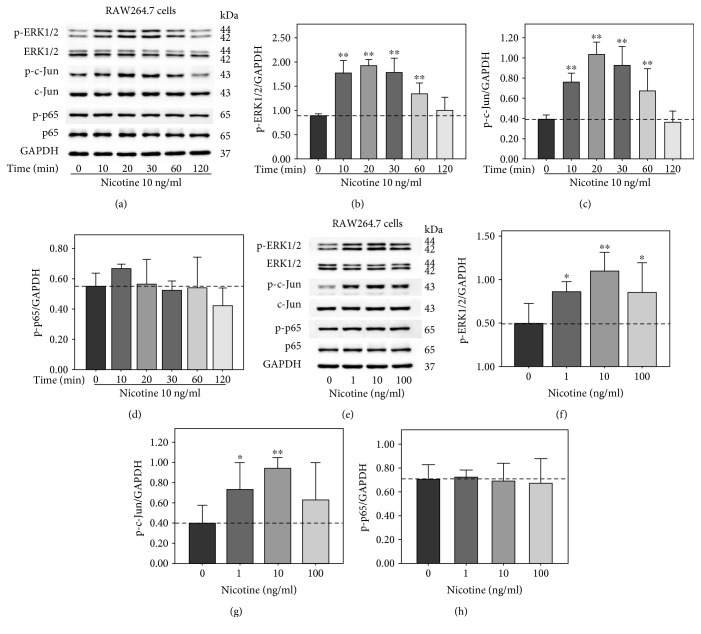
Nicotine stimulated the phosphorylation of extracellular signal-regulated kinase1/2 (ERK1/2) and c-Jun in RAW264.7 cells. (a) RAW264.7 cells were treated with 10 mg/ml nicotine for 0, 10, 20, 30, 60, and 120 min. (e) RAW264.7 cells were exposed to nicotine for 30 min at the concentrations of 0, 1, 10, and 100 mg/ml. Cell lysates were collected and protein levels of p-ERK1/2, ERK1/2, p-c-Jun, c-Jun, p-p65, and p65 were measured by Western blot. The band optical density values (means ± SD) of p-ERK1/2 (b, f), p-c-Jun (c, g), and p-p65 (d, h) were evaluated using ImageJ software, with all experiments being analyzed as three different independent experiments and GAPDH used as an internal control. ^∗^*p* < 0.05 and ^∗∗^*p* < 0.01 versus controls.

Nicotine is the main ingredient of cigarette smoke, and plasma concentration of nicotine is rapidly increased after a single cigarette, ranging from 10–50 ng/ml [20]. To investigate the underlying mechanisms of nicotine-induced AAA, RAW264.7 cells were exposed to 10 ng/ml nicotine for 0, 10, 20, 30, 60, and 120 min. Western blot analysis (Figure 1(a)) revealed that 10 ng/ml nicotine resulted in significant increase in the phosphorylation of ERK1/2 and c-Jun from 10 min to 60 min; however, nicotine had no effect on p65 phosphorylation. Then, RAW264.7 cells were treated with nicotine for 30 min at the concentrations of 0, 1, 10, and 100 ng/ml. As seen in Figure 1(e), the phosphorylation of ERK1/2 and c-Jun was upregulated by nicotine, whereas the protein levels of p-p65 remained unchanged. These data suggest that nicotine may exert biological effects via modulating ERK1/2 and c-Jun signaling pathway in RAW264.7 cells.

### 3.2. U0126 Abolished Nicotine-Induced Activations of ERK1/2 and c-Jun and Expression of MMP-9, MMP-2, MCP-1, and RANTES in RAW264.7 Cells

**Figure 2:**
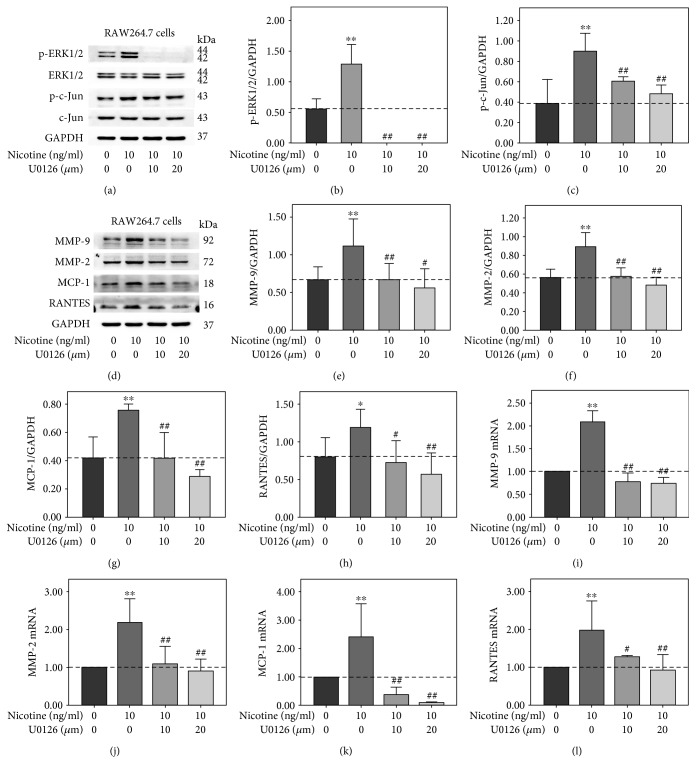
U0126 attenuated nicotine-induced activations of extracellular signal-regulated kinase1/2 (ERK1/2) and c-Jun and upregulation of matrix metalloproteinase- (MMP-) 9, MMP-2, monocyte chemotactic protein- (MCP-) 1, and regulated upon activation normal T cell expressed and secreted (RANTES) in RAW264.7 cells. (a) RAW264.7 cells were pretreated with 10 *μ*m and 20 *μ*m U0126 for 30 min prior to 10 ng/ml nicotine exposure for 30 min. (d) RAW264.7 cells were pretreated with 10 *μ*m and 20 *μ*m U0126 for 30 min prior to 10 ng/ml nicotine exposure for 3 h. (b, c, e–h) The intensity of protein bands (means ± SD) in the Western blot were quantified by using ImageJ software and normalized to GAPDH. Representative results from three independent experiments are shown. (i) MMP-9, (j) MMP-2, (k) MCP-1, and (l) RANTES mRNA levels were determined by real-time reverse transcription polymerase chain reaction. All experiments were analyzed as triplicate independent experiments. ^∗^*p* < 0.05 and ^∗∗^*p* < 0.01 versus controls; ^#^*p* < 0.05 and ^##^*p* < 0.01 versus the group treated with nicotine.

In order to further determine whether ERK1/2 and c-Jun signaling pathway is involved in nicotine-induced expression of MMP-9, MMP-2, MCP-1, and RANTES, RAW264.7 cells were pretreated with 10 *μ*m and 20 *μ*m U0126 for 30 min prior to 30 min or 3 h nicotine exposure at the concentration of 10 ng/ml. Nicotine-induced activation of ERK1/2 and c-Jun was significantly abolished by U0126 (Figure 2(a)). Also, nicotine-induced upregulation of MMP-9, MMP-2, MCP-1, and RANTES was remarkably decreased shown in Figure 2(d). Moreover, quantitative RT-PCR showed that U0126 inhibited nicotine-induced MMP-9, MMP-2, MCP-1, and RANTES mRNA expression (Figures 2(i), 2(j), 2(k), and 2(l)). The results indicate that nicotine induces upregulation of MMP-9, MMP-2, MCP-1, and RANTES through activating ERK1/2/c-Jun pathway in RAW264.7 cells.

### 3.3. Nicotine Increased Phosphorylation of c-Jun in MOVAS Cells

**Figure 3:**
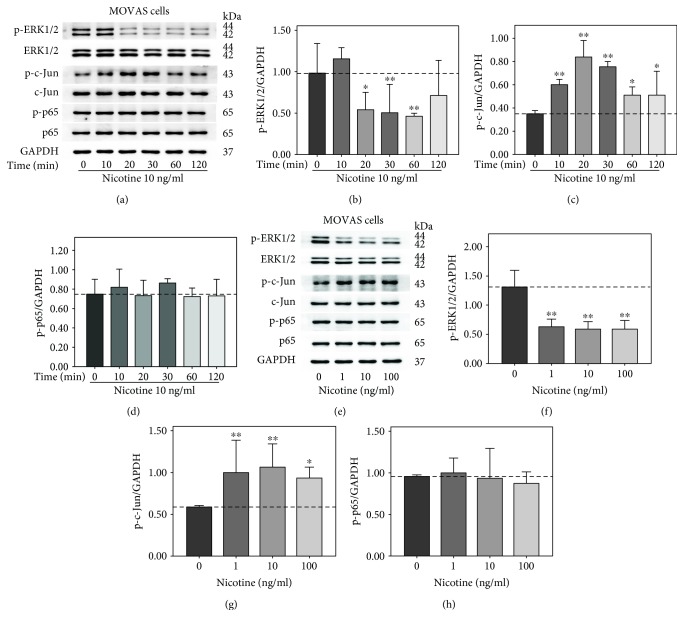
Nicotine increased the phosphorylation of c-Jun in MOVAS cells. (a) MOVAS cells were exposed to 10 ng/ml nicotine for 0, 10, 20, 30, 60, and 120 min. (e) MOVAS cells were treated with nicotine for 30 min at the concentrations of 0, 1, 10, and 100 ng/ml. The expression of phosphorylated extracellular signal-regulated kinase1/2 (p-ERK1/2), ERK1/2, p-c-Jun, c-Jun, p-p65, and p65 were analyzed with Western blot. (b–d, f–h) Densitometric analysis of protein bands was performed via ImageJ software. GAPDH was utilized as an internal control, and all experiments were analyzed as three different independent experiments. ^∗^*p* < 0.05 and ^∗∗^*p* < 0.01 versus controls.

We also explored whether nicotine had any effect on the ERK1/2, c-Jun, and p65 in MOVAS cells. Similarly, MOVAS cells were treated with 10 ng/ml nicotine for 0, 10, 20, 30, 60, and 120 min. As shown in Figure 3(a), nicotine-induced phosphorylation of c-Jun markedly increased at 10 min and 20 min and then gone down from 30 min to 120 min. In contrast, nicotine suppressed ERK1/2 phosphorylation and had no effect on the activation of p65. Then, MOVAS cells were exposed to nicotine at various concentrations (0, 1, 10, and 100 ng/ml) for 30 min. The phosphorylation of c-Jun was significantly elevated at all three concentrations, whereas ERK1/2 phosphorylation was inhibited (Figure 3(e)). The activation of p65 was not affected by nicotine at different concentration. These data reveal that c-Jun may be an important transcription factor involving in nicotine-induced biological effects in MOVAS cells. Inconsistent with RAW264.7 cells, MOVAS cells treated with nicotine exhibited contrary tendency between ERK1/2 and c-Jun. It is worth mentioning that Cho et al. had showed that U0126 had no effect on TNF*α*-induced activation of c-Jun in VSMCs [21]. Therefore, we hypothesized that nicotine evoked different signal transduction pathway to play biological roles in different cell types.

### 3.4. PNU-282987 Suppressed Nicotine-Stimulated Activation of ERK1/2 and c-Jun and Upregulation of MMP-9, MMP-2, MCP-1, and RANTES in RAW264.7 Cells

**Figure 4:**
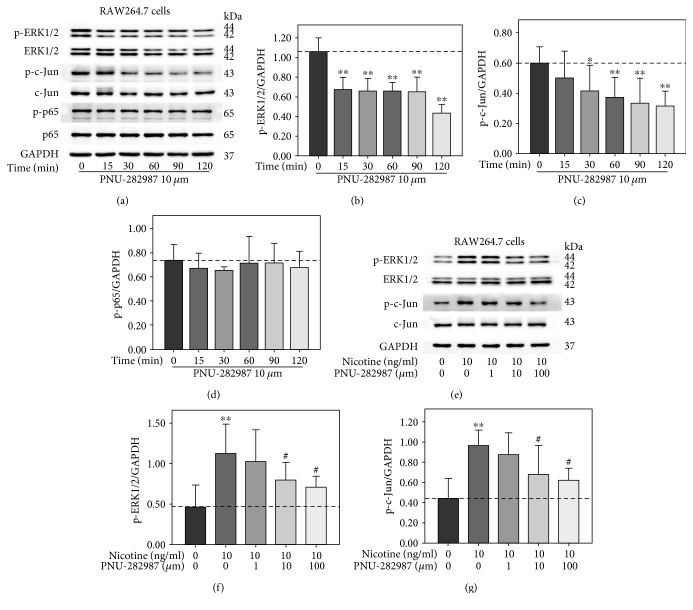
PNU-282987 suppressed nicotine-stimulated activation of extracellular signal-regulated kinase1/2 (ERK1/2) and c-Jun in RAW264.7 cells. (a) RAW264.7 cells were treated with 10 *μ*m PNU-282987 for 0, 15, 30, 60, 90, and 120 min. (e) RAW264.7 cells were pretreated with 10 *μ*m PNU-282987 for 60 min prior to 10 ng/ml nicotine exposure for 30 min. Protein levels of p-ERK1/2, ERK1/2, p-c-Jun, c-Jun, p-p65, and p65 were measured by Western blot, and the intensity of protein bands (means ± SD) (b–d, f, g) were assessed using ImageJ software, with all experiments being analyzed as three different independent experiments and GAPDH used as an internal control. ^∗^*p* < 0.05 and ^∗∗^*p* < 0.01 versus controls; ^#^*p* < 0.05 and ^##^*p* < 0.01 versus the group treated with nicotine.

**Figure 5:**
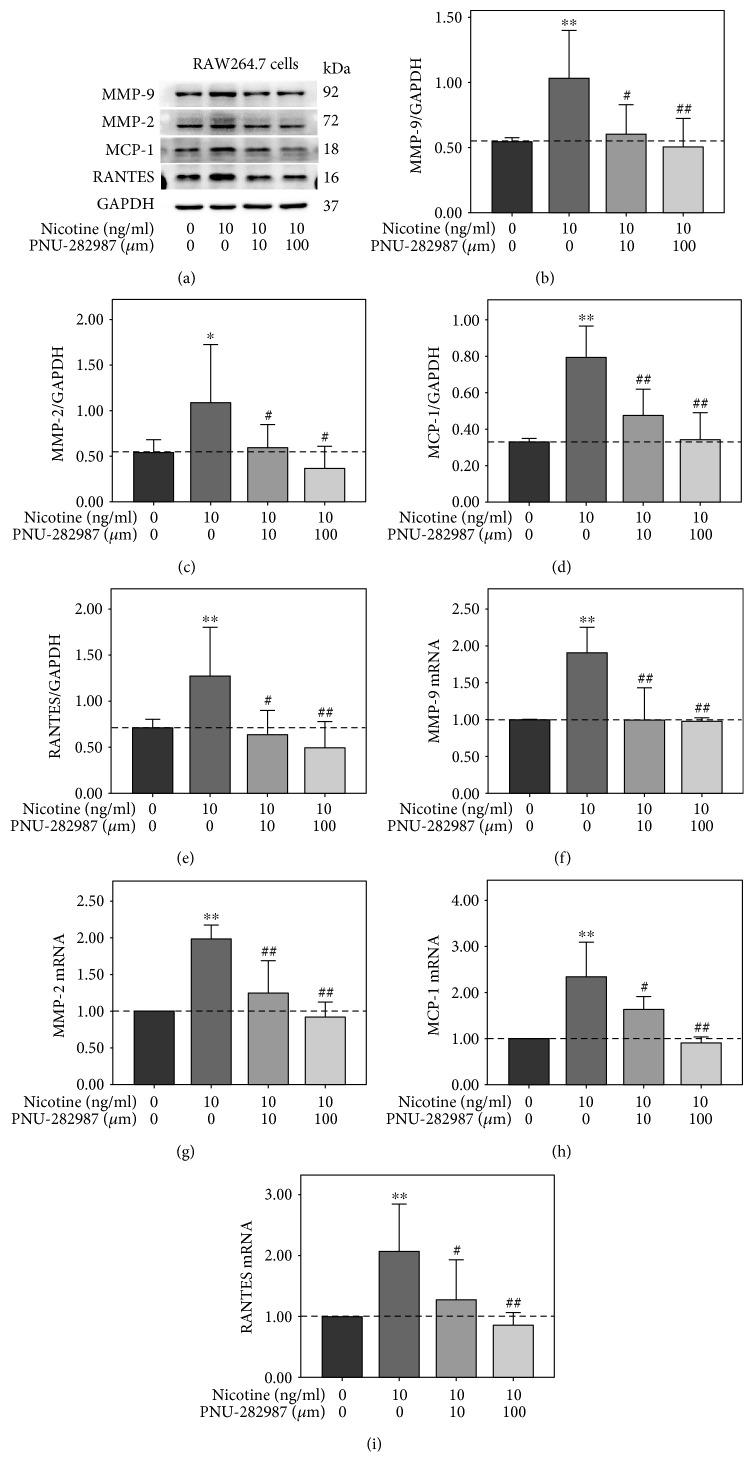
PNU-282987 abrogated nicotine-induced upregulation of matrix metalloproteinase- (MMP-) 9, MMP-2, monocyte chemotactic protein- (MCP-) 1, and regulated upon activation normal T cell expressed and secreted (RANTES) in RAW264.7 cells. (a) RAW264.7 cells were pretreated with 10 *μ*m PNU-282987 for 60 min prior to 10 ng/ml nicotine exposure for 3 h. The band optical density values (means ± SD) of (b) MMP-9, (c) MMP-2, (d) MCP-1, and (e) RANTES in the Western blot were quantified by using ImageJ software and normalized to GAPDH. Representative results from three independent experiments are shown. (f) MMP-9, (g) MMP-2, (h) MCP-1, and (i) RANTES mRNA levels were examined by real-time reverse transcription polymerase chain reaction. All experiments were analyzed as triplicate independent experiments. ^∗^*p* < 0.05 and ^∗∗^*p* < 0.01 versus controls; ^#^*p* < 0.05 and ^##^*p* < 0.01 versus the group treated with nicotine.

The cholinergic anti-inflammatory pathway is composed of the efferent vagus nerve, the neurotransmitter acetylcholine, and *α*7-nAChR [17]. Recently, the potential role vagus nerve stimulation in cardiovascular disease has emerged [18]. To determine the effect of *α*7-nAChR on nicotine-stimulated expression of MMPs and inflammation cytokines, cells were stimulated in the presence of the *α*7-nAChR selective agonist. In the first experiment, RAW 264.7 cells were exposed to 10 *μ*m PNU-282987 for 0, 15, 30, 60, 90, and 120 min. Western blot analysis showed that PNU-282987 markedly suppressed the phosphorylation of ERK1/2 and c-Jun (Figure 4(a)). Nevertheless, p65 phosphorylation had a descending tendency without statistical significance. Then, RAW264.7 cells were pretreated with PNU-282987 at various concentrations (0, 1, 10, and 100 *μ*m) for 30 min prior to 10 ng/ml nicotine exposure for 30 min or 3 h. As shown in Figure 4(e), nicotine-induced activation of ERK1/2 and c-Jun was abolished by PNU-282987 at the concentrations of 10 and100 *μ*m. At the same concentrations, PNU-282987 suppressed nicotine-stimulated upregulation of MMP-9, MMP-2, MCP-1, and RANTES (Figure 5(a)). In addition, quantitative RT-PCR showed that PNU-282987 also attenuated nicotine-induced MMP-9, MMP-2, MCP-1, and RANTES mRNA expression (Figures 5(f), 5(g), 5(h), and 5(i)). These results suggest that *α*7-nAChR agonist inhibits nicotine-induced upregulation of MMP-9, MMP-2, MCP-1, and RANTES through modulating ERK1/2/c-Jun signaling in RAW264.7 cells.

### 3.5. PNU-282987 Attenuated Nicotine-Induced Activations of c-Jun and Expression of MMP-9, MMP-2, MCP-1, and RANTES in MOVAS Cells

**Figure 6:**
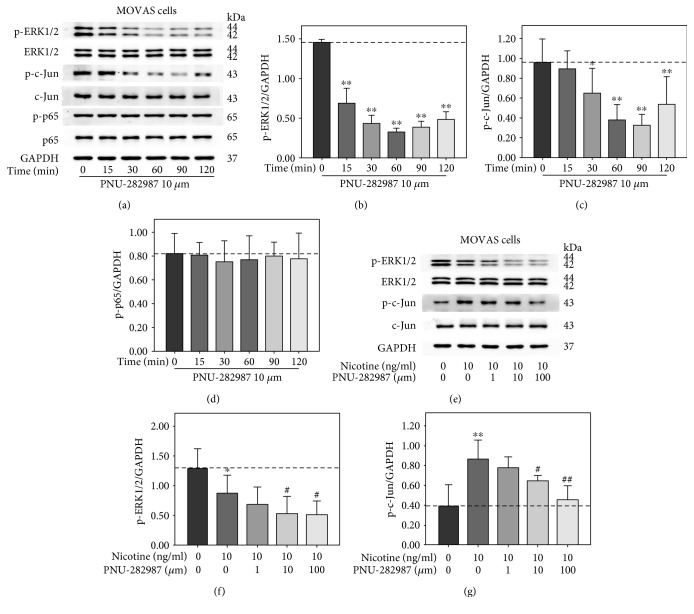
PNU-282987 inhibited nicotine-stimulated activation of c-Jun in MOVAS cells. (a) MOVAS cells were exposed to 10 *μ*m PNU-282987 for 0, 15, 30, 60, 90, and 120 min. (e) MOVAS cells were pretreated with 10 *μ*m PNU-282987 for 60 min prior to 10 ng/ml nicotine exposure for 30 min. Cell lysates were collected, and protein levels of phosphorylated extracellular signal-regulated kinase1/2 (p-ERK1/2), ERK1/2, p-c-Jun, c-Jun, p-p65, and p65 were measured by Western blot. (b–d, f, g) Densitometric analysis of protein bands was performed via ImageJ software, with all experiments being analyzed as three different independent experiments and GAPDH used as an internal control. ^∗^*p* < 0.05 and ^∗∗^*p* < 0.01 versus controls; ^#^*p* < 0.05 and ^##^*p* < 0.01 versus the group treated with nicotine.

**Figure 7:**
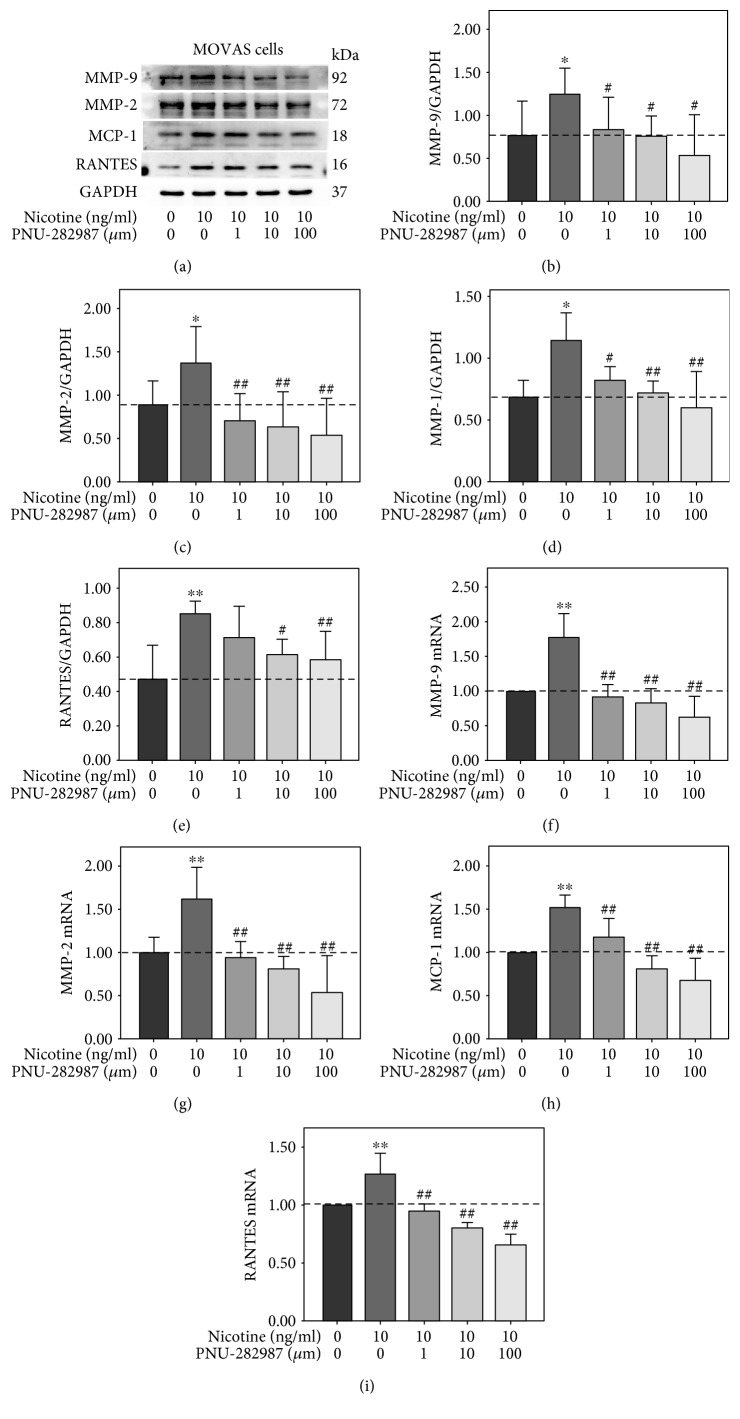
PNU-282987 downregulated nicotine-induced expression of matrix metalloproteinase- (MMP-) 9, MMP-2, monocyte chemotactic protein- (MCP-) 1, and regulated upon activation normal T cell expressed and secreted (RANTES) in MOVAS cells. (a) MOVAS cells were pretreated with 10 *μ*m PNU-282987 for 60 min prior to 10 ng/ml nicotine exposure for 3 h. The band optical density values (means ± SD) of (b) MMP-9, (c) MMP-2, (d) MCP-1, and (e) RANTES in the Western blot were quantified by using ImageJ software and normalized to GAPDH. Representative results from three independent experiments are shown. (f) MMP-9, (g) MMP-2, (h) MCP-1, and (i) RANTES mRNA levels were determined by real-time reverse transcription polymerase chain reaction. All experiments were analyzed as triplicate independent experiments. ^∗^*p* < 0.05 and ^∗∗^*p* < 0.01 versus controls; ^#^*p* < 0.05 and ^##^*p* < 0.01 versus the group treated with nicotine.

In the second experiment, MOVAS cells were treated with 10 *μ*m PNU-282987 for 0, 15, 30, 60, 90, and 120 min. Western blot analyses indicated that ERK1/2 and c-Jun phosphorylation was remarkably decreased at different time points, whereas the phosphorylation of p65 was not changed (Figure 6(a)). Then, MOVAS cells were pretreated with PNU-282987 at various concentrations (0, 1, 10, and 100 *μ*m) for 60 min prior to 10 ng/ml nicotine treatment for 30 min or 3 h. As shown in Figure 6(e), nicotine-induced activation of c-Jun was attenuated by PNU-282987 at the concentrations of 10 and 100 *μ*m. The inhibition of ERK phosphorylation by nicotine was further enhanced in MOVAS cells pretreated with PNU-282987. Moreover, PNU-282987 suppressed nicotine-stimulated excretion of MMP-9, MMP-2, MCP-1, and RANTES from MOVAS cells (Figure 7(a)). Also, quantitative RT-PCR showed that PNU-282987 also reduced nicotine-induced mRNA expression of MMP-9, MMP-2, MCP-1, and RANTES shown in Figures 7(f), 7(g), 7(h), and 7(i). These results suggest that *α*7-nAChR agonist inhibits nicotine-induced expression of MMP-9, MMP-2, MCP-1, and RANTES via c-Jun in MOVAS cells.

## 4. Discussion

In the present study, we demonstrated for the first time that stimulation of *α*7-nAChR suppressed nicotine-induced upregulation of inflammatory cytokines and MMP. MCP-1 and RANTES, as representative of CC chemokine family, had also been demonstrated involvement in AAA development [7, 22] and thought to play greater roles than other chemokines [23]. MCP-1 promoted macrophage infiltration, increased the MMP-9 expression in SMCs, and induced apoptosis of SMCs within AAA, either through a direct mechanism or via activation of macrophages [22, 24, 25]. RANTES, similarly to MCP-1, acts on multiple immune cells and plays an important role in chronic and acute inflammation [26]. MMP-9 and MMP-2, which degrade the extracellular matrix, work in concert to form AAA, and both MMP-9 and MMP-2 knockout mice are resistant to aneurysm development [27]. Inflammation and extracellular matrix degradation play crucial roles in AAA formation. Therefore, it is likely that suppression of MCP-1, RANTES, MMP-9, and MMP-2 through activation of *α*7-nAChR resulted in the attenuation of nicotine-induced AAA development.

The molecular mechanisms associated *α*7-nAChR agonist with suppression of nicotine-induced AAA formation may be ERK1/2 and AP-1- (c-Jun-) mediated signaling pathway in macrophages and SMCs. ERK1/2 belongs to mammalian mitogen-activated protein kinase (MAPK) family, which play a crucial role in various cellular responses, including cell proliferation, differentiation, and survival [28, 29]. Transcription factor AP-1 family comprises multiple Jun (c-Jun, JunB, and JunD) and Fos (c-Fos, FosB, Fral, and Fra2) members [30]. AP-1 had been found involvement in several physiological and pathological cellular processes including proliferation, inflammation, differentiation, growth, apoptosis, cell migration, and transformation [30, 31]. This study showed that nicotine increased expression of MCP-1, RANTES, MMP-9, and MMP-2 through activating ERK1/2/AP-1 (c-Jun) signaling pathway in RAW264.7 cells and AP-1 (c-Jun) in MOVAS cells. These results indicated that AP-1 (c-Jun) might play an important role in nicotine-induced AAA formation, which is further confirmed by the report that AP-1 (c-Jun) was remarkably elevated in human AAA [32]. Stimulation of *α*7-nAChR by PNU-282987 significantly inhibited nicotine-induced activation of ERK1/2 and AP-1 (c-Jun) in RAW264.7 and MOVAS cells and then suppressed nicotine-stimulated upregulation of MCP-1, RANTES, MMP-9, and MMP-2. Interestingly, nicotine might activate c-Jun through different signaling pathways in RAW264.7 and MOVAS cells. Our previous study demonstrated that nicotine activated and PNU-282987 inhibited JNK signaling in MOVAS cells, which could be another upstream pathway of c-Jun [33]. The elucidation of such signaling pathways will reveal novel molecular targets that may provide a new therapeutic strategy for AAA. It needed to mention that nicotine could not evoke the activation of NF-*κ*B p65 in this study. Although it is well known that NF-*κ*B regulates expression of multiple inflammatory cytokines, it may play little role in the process of nicotine-induced secretion of MCP-1 and RANTES.

The nAChRs are a family of ligand-gated ion channel receptors composed of 17 subunits *α*1–*α*10, *β*1–*β*4, *γ*, *δ*, and *ε* [34]. In addition to the central and peripheral nervous system, the nAChRs have been identified in vascular tissue and immune cells [34, 35]. The different subunit combinations result in functionally diverse nAChR subtypes that have different ligand affinity, cation permeability, and signaling [36, 37]. The involvement of nAChR in nicotine-induced expression of inflammatory cytokines and MMP and development of AAA remains unknown. Nevertheless, accumulating evidence points towards a protective role for *α*7-nAChR in inflammation-based cardiovascular diseases. Cheng et al. showed that *α*7-nAChR activation reduced the expression of TNF*α* and IL-6 and alleviated viral myocarditis [38]. Treatment with PNU-282987 inhibited ADP-induced platelet aggregation in human platelets, and hematopoietic *α*7-nAChR deficiency increased number of peritoneal leukocytes and expression of inflammatory mediators by both peritoneal leukocytes (TNF*α* and CRP) and the spleen (TNF*α*) [39], both thought to be important risk factors in atherosclerotic lesion development [40]. Stimulation of *α*7-nAChR by AR-R1779 attenuates atherogenesis in apolipoprotein E-deficient mice treated with AngII possibly through an anti-inflammatory effect and reduction of blood pressure and lipid levels [41].

In contrast to nicotine that activates multiple nAChRs subtypes, PNU-282987 is a selected agonist for *α*7-nAChR. Thus, we inferred that distinct effects of nicotine and PNU-282987 might be mediated by differential nAChR subtypes. In accordance with the assumption, a recent study by de Moura and McMahon showed that PNU-282987 failed to substitute for the nicotine discriminative stimulus in male C57BL/6J mice [42]. Another study showed that nicotine-induced catecholamine release from the adrenal glands was modulated by *α*3*β*4nAChR, but not by *α*7nAChR [43].

## 5. Conclusion

Taken together, *α*7-nAChR agonist inhibits nicotine-induced upregulation of inflammatory cytokines and MMP through modulating ERK1/2/AP-1 signaling in RAW264.7 cells and AP-1 in MOVAS cells. *α*7-nAChR agonist is expected to be a new therapeutic strategy for AAA. However, more studies, experimental and clinical ones, are necessary to gain further insights into the function and signaling of nAChRs and offer rational therapeutic strategies.
